# Assessing the impact of the “malaria supporters project” intervention to malaria control in the Brazilian Amazon: an interrupted time-series analysis

**DOI:** 10.1186/s12936-023-04706-z

**Published:** 2023-09-16

**Authors:** Klauss Kleydmann Sabino Garcia, Seyi Soremekun, Christian Bottomley, Amanda Amaral Abrahão, Cristiano Barreto de Miranda, Chris Drakeley, Walter Massa Ramalho, André M. Siqueira

**Affiliations:** 1 Nucleus of Tropical Medicine, University of Brasilia, Brasilia, Federal District Brazil; 2 Department of Infection Biology, Faculty of Infectious and Tropical Diseases, University of London-London School of Hygiene & Tropical Medicine, London, UK; 3 MRC International Statistics and Epidemiology Group, University of London-London School of Hygiene & Tropical Medicine, London, UK; 4 Secretary of Health and Environment Surveillance, Ministry of Health, Brasilia, Federal District Brazil; 5 School of Public Health, University of São Paulo, São Paulo, São Paulo Brazil; 6 FIOCRUZ, Evandro Chagas National Institute of Infectology, Rio de Janeiro, Rio de Janeiro Brazil

**Keywords:** Malaria, Epidemiology, Public Health, Control, Interrupted time series, Brazil

## Abstract

**Background:**

In 2021, Brazil was responsible for more than 25% of malaria cases in the Americas. Although the country has shown a reduction of cases in the last decades, in 2021 it reported over 139,000 malaria cases. One major malaria control strategy implemented in Brazil is the “Malaria Supporters Project”, which has been active since 2012 and is directed to municipalities responsible for most Brazil’s cases. The objective of this study is to analyse the intervention effect on the selected municipalities.

**Methods:**

An ecological time-series analysis was conducted to assess the “Malaria Supporters Project” effect. The study used data on Annual Parasitic Incidence (API) spanning the period from 2003 to 2020 across 48 intervention municipalities and 88 control municipalities. To evaluate the intervention effect a Prais–Winsten segmented regression model was fitted to the difference in malaria Annual Parasitic Incidence (API) between control and intervention areas.

**Results:**

The intervention group registered 1,104,430 cases between 2012 and 2020, a 50.6% reduction compared to total cases between 2003 and 2011. In 2020 there were 95,621 cases, 50.4% fewer than in 2011. The number of high-risk municipalities (API > 50 cases/1000) reduced from 31 to 2011 to 17 in 2020. The segmented regression showed a significant 42.0 cases/1000 residents annual decrease in API compared to control group.

**Conclusions:**

The intervention is not a silver bullet to control malaria, but it has reduced API in locations with high malaria endemicity. Furthermore, the model has the potential to be replicated in other countries with similar epidemiological scenarios.

**Supplementary Information:**

The online version contains supplementary material available at 10.1186/s12936-023-04706-z.

## Background

The WHO estimates that malaria has caused more than 247 million cases worldwide in 2021, with an incidence rate of 59 cases per 1000 people at risk. In the Americas, malaria estimates are over 600,000 cases with 4 cases per 1000 people at risk. Brazil is the second country with the most malaria cases in the Americas, responsible for more the 25% of the region cases [1]. In Brazil, malaria is a serious public health issue that impacts the population socially and economically. The disease has undergone historical changes throughout the 20th century with cases increase in the entire country. In the second half of the 20th century, malaria cases in Brazil became concentrated in the Brazilian Amazon region and increased until 2005, when over 600,000 cases were recorded [2, 3]. In 2010, it registered around 326,286 autochthonous cases, in 2015 it registered 138,270. In 2017 and 2018, Brazil presented a general increase in malaria cases, registering over 184,000 autochthonous cases in each year. After 2018, Brazil started to reduce malaria cases again, registering less than 139,000 autochthonous cases in 2021 [4].

The Brazilian Ministry of Health (BMoH) strategies to control and prevent malaria are operated through the National Programme for the Control and Prevention of Malaria (PNCM), which is composed of surveillance centers in the federal government, in states and municipal levels. The BMoH employs WHO-recommended strategies like timely cases identification, providing free treatment fast [4], use of insecticides, distribution and installation of insecticide-impregnated mosquito nets, and federal support for municipalities [5, 6].

Recently in 2022, the BMoH published the National Plan for Eliminating Malaria until 2035 in compliance with the Sustainable Development Goals [7]. Among the strategies cited to be used as a tool to elimination is the “Municipal Supporters for Malaria Control in the Brazilian Amazon region” (Malaria Supporters Project).

The Malaria Supporters Project was developed and implemented to specifically address malaria strategy for prevention and control in the hardest hit municipalities responsible for 80% of all Brazil’s cases [8, 9]. Between 2010 and 2012, Brazil implemented the “Expanding Access to Malaria Prevention and Control Measures for Vulnerable Populations in the Brazilian Amazon” project, funded by The Global Fund. The project aimed to strengthen local health services to promote better malaria control actions with use of epidemiological intelligence. The priority municipalities for malaria control had advisers responsible for epidemiological monitoring, training the local team in epidemiological analysis and strengthening local management in the prevention and control of the disease. The main purpose was to ensure the sustainability of actions through technical strengthening and the generation of autonomy for municipal teams.

In view of the success of the Global Fund Project and the actions carried out in it, the Brazilian Ministry of Health decided to continue the advisory actions. Thus, in 2012, it implemented the Municipal Malaria Supporters Project [10]. The project is a potential model that could be scaled-up and replicated elsewhere, and it is a result from a partnership between the PNCM/BMoH and the Oswaldo Cruz Foundation (Fiocruz). The Project’s objective is to strengthen local malaria surveillance services based on a work methodology, that could be continued by the municipalities even after the Project being interrupted [4].

The Project directs Supporters with experience and expertise in public health and technical capacity to act in epidemiological surveillance and health planning actions. They represent active support of the PNCM/BMoH in the municipal and state planning process to fulfill PNCM recommendations [11], and it is recognized by the Pan American Health Organization (PAHO) as fundamental in responding to high burden municipalities, towards malaria elimination [10].

According to the PNCM, the Project is currently active (2023) with a budget of 6.4 million R$ (~ 1.2 million US$) for execution between 2019 and 2022 (information updated in July 2021). Between 2012 and 2020, the Project had an accumulated amount of 38.2 million R$ (~ 7.1 million US$) and was active in 48 municipalities (Source: BMoH, Additional file 1).

In this study, a controlled interrupted time series analysis is employed to evaluate the impact of the “Malaria supporters project” intervention on malaria Annual Parasitic Incidence (API) in the supported municipalities between 2012 and 2020.

## Methods

### Study design and data sources

This is an ecological time series study using counts of malaria cases per year spanning the period from 2003 to 2020. Data was obtained from the Epidemiological Surveillance System for Malaria (Sivep-Malaria) established by the BMoH in 2003. Sivep-malaria data was updated in January 2023 and provided by the BMoH (protocol #25072.007957/2021-05, and #25072.004310/2023-85).

Information about the timing of the intervention was also provided by the PNCM/BMoH, through the Information Access Portal (protocol #25072.020758/2021-84, and #25072.003428/2023-96) (Additional file 1).

### Study site and period

The analysis involved data from 48 “supported” municipalities where the intervention was implemented starting in 2012 and 88 neighbouring control municipalities where it was not. A list of municipalities in the intervention and control groups is provided in Additional file 2. The study timeline is from 2003 to 2020, with the intervention being implemented in 2012. The analysis of epidemiological data was conducted during the intervention period of 2012 to 2020 (9 years), with a comparative focus on the preceding 9 years (2003–2011) when the intervention was not yet in effect. Both intervention and control municipalities are located in the states of Acre, Amazonas, Roraima, Rondônia, Pará, and Amapá all in the Amazon region of Brazil. The climate in the Amazon region is hot and humid [12] and the environmental conditions are extremely favourable for the proliferation of mosquitoes. The main malaria vector in the region is the mosquito *Anopheles darlingi* [13, 14].

### Intervention characterization

The intervention was rolled out in 2012, after the completion of the international technical cooperation between the Brazilian government and the Global Fund for malaria actions. After the cooperation, the Brazilian government committed to continue using control strategies to mitigate the impact of malaria in the Amazon region of Brazil (information provided by the BMoH and available in Additional file 1).

Its execution takes place via the Decentralized Term of Execution between the BMoH and Fiocruz, with national funding. The supporters develop their work routines together with local malaria prevention, control, and elimination teams. The supporters’ actions should contribute to improve quality of diagnosis, treatment, vector control, health situation analysis, and health education actions. The aim is to improve malaria epidemiological indicators, and to strengthen the local service, passing on to the municipal health teams a work methodology that can be maintained in a sustainable way [10].

For the municipalities selection to receive the project (intervention) different aspects are evaluated, such as: the total number of new autochthonous infections in the municipality; the period in which the municipality remained a priority municipality; the actions of prevention, control and malaria elimination undergone; epidemiological scenario; and operational capacity of local support to the professional. Priority is given to the areas of greatest endemicity for malaria.

The supporters are required to possess a university degree in health-related disciplines such as public health, medicine, biology, among others. While postgraduate qualifications are not obligatory, they are considered desirable in the selection process. Moreover, they should demonstrate proficiency in data tabulation, analysis, and interpretation, backed by a minimum of 3 years of professional engagement with health surveillance services. Additionally, a substantial track record of at least 5 years within the specified domains is essential.

The supporters have continuous communication with the PNCM and Malaria State Programmes. Their activities are monitored through the analysis of technical activities reports and their annual work plan. Additionally, there are moments to discuss strategies and activities in online and face-to-face meetings, which helps on problems solving and better communication.

The supporters act as epidemiological intelligence agents, being a strong communication channel between the federal, state, and municipal malaria control programmes. The central idea is that they train the municipal malaria control programme to work on control actions and on epidemiology surveillance, as recommended by the PNCM/BMoH.

Before starting their activities in the municipalities, the supporters receive training from the PNCM/BMoH, and in some situations, they also receive training from the state epidemiological teams. The training takes 40 to 80 h (5 to 10 days), and they are gathered at the BMoH headquarters to participate in workshops with PNCM technicians.

The supporters receive instructions on how to effectively manage and guide local administrations, especially when dealing with potential disagreements. The training emphasizes the core components of the PNCM programme, encompassing epidemiological surveillance, programme foundations, and importance, along with everyday malaria control scenarios. It covers crucial aspects including: Health information systems, underlining their role in disease control and their functionalities; Health education, social control, and management tools, underscoring their significance and potential for disease control; vector control, with a focus on viable local scenario-based applications; appropriate diagnostic and treatment approaches in line with BMoH guidelines; and effective management of the public budget and supplies, complete with instructions for meticulous monitoring of diagnostic, treatment, and vector control resources.

### Control group characterization

The selection of all neighbouring municipalities as a control group considered comparable attributes shared with the intervention group municipalities such as geographical and demographics factors, epidemiological trends and behaviours, while also accounting for potential confounding variables [15, 16]—such as the presence of additional malaria control tools, such as insecticides and other entomological measures that assumedly affect both control and intervention groups similarly.

Notably, the distinguishing factor lies in the absence of intervention exposure within the control group municipalities throughout the designated period [15, 16]. Consequently, neighbouring municipalities were chosen as the control group due to their proximity, ensuring exposure to analogous environmental, climatic, and socio-economic conditions that could potentially affect malaria incidence.

### Statistical analysis

The main outcome was the malaria annual parasitic incidence (API). It was calculated by dividing the total number of new malaria infections (all ages) per year, excluding recurrences, by estimates of population size obtained from the Brazilian Institute of Geography and Statistics [17]. The API is a crucial indicator to measure intensity of malaria in a population over a specific period. To calculate the API, “date of case notifications” and “location of probable infection” variables were used from the database (Sivep-Malaria) provided by the BMoH.

Supported municipalities were classified according to their API, and the proportion at each level compared before and after the introduction of the intervention. API is a measure of malaria risk used by the PNCM/BMoH that stratifies risk into four levels: very low risk (< 1.0 case/1000 residents); low risk (1.0–9.9 cases/1000 res.); medium risk (10.0–50 cases/1000 res.) and high risk (API > 50 cases/1000 res.) [4].

To estimate the impact of the intervention a controlled interrupted time-series (cITS) was conducted. An interrupted time-series analysis encompasses various methods designed to handle severe fluctuations in data. When dealing with volatile or erratic patterns, several approaches can be employed to understand the impact of extreme fluctuations. It is also capable of identifying time trends, seasonality and to correct data autocorrelation. Therefore, the decrease trend prior to the intervention period is analysed and compared to intervention period data [16]. The control analysis subtracts the control series from the intervention series and analyses the resulting differences. The subtraction allows to correct data estimates by minimizing the effect of common trends, seasonalities and potential confounding [18–20]. Specifically, a linear regression model was constructed with the API differences serving as the outcome, and a binary indicator variable representing the intervention period serving as an explanatory variable. The Prais-Winsten method was used to account for autocorrelation in the differences [21, 22].

All analyses were done using R (version 4.2.1) [23] and the “prais” package was used to implement the Prais–Winsten method (Code in Additional file 3) [24]. A p-value cut-off of 0.05 was employed to establish statistical significance.

### Ethical considerations

This research did not require approval by ethics committees according to the Brazilian National Health Council resolution no. 466/2012 because it does not use sensitive or nominal data for its performance, only estimates of case counts by municipality of infection. All information presented in this work is of a public nature and was provided by official means of access to information from the BMoH.

## Results

### Descriptive patterns of malaria in the study region

The 48 supported municipalities reported 3,341,092 malaria cases from 2003 to 2020 (annual average: 185,616; minimum: 95,621 in 2020, and maximum: 348,646 in 2005). Between 2003 and 2011 (pre-intervention) there were 2,236,662 new malaria infections and between 2012 and 2020 (post-intervention) there were 1,104,430 new infections, a reduction of 50.6% compared to the first period. The control group reported 1,200,477 cases between 2003 and 2020 (annual average: 66,693; minimum: 21,708 in 2016, and maximum: 159,425 in 2005). Between 2003 and 2011 the control group registered 889,254 cases and from 2012 to 2020 it registered 311,223 cases, a reduction of 65.0% compared to the first period.

**Fig. 1:**
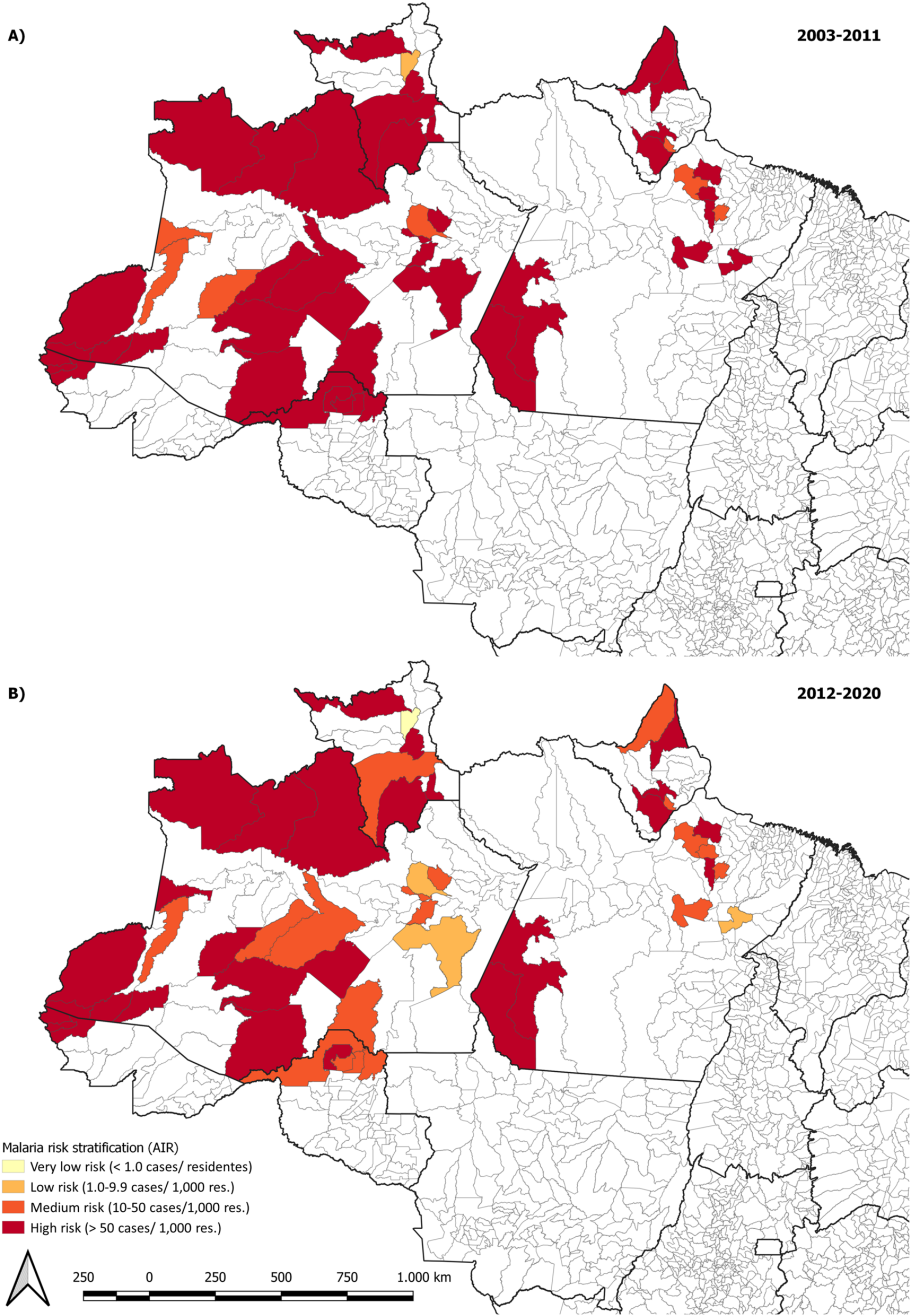
Malaria risk stratification in supported municipalities, 2003–2020. **A** Risk stratification based on the Annual Parasitic Incidence per 1000 residents between 2003 and 2011; **B** risk stratification based on the Annual Parasitic Incidence per 1000 residents between 2012 and 2020 (Source: Sivep-Malaria—Ministry of Health)

Prior to the intervention in the 48 municipalities 40 (83.3%) of them were high-risk municipalities based in the average API between 2003 and 2011 (API over 50 cases/1000 res.) and the average API between 2012 and 2020 reflected a reduction of 35.0% (N = 26) in the total of high-risk municipalities (Fig. 1). The control groups presented 36 (40.9%) high-risk municipalities from 2003 to 2011 (based on the average API), and 10 (11.3%) from 2012 to 2020, a reduction of 72.2% on the number of high-risk municipalities.

**Fig. 2:**
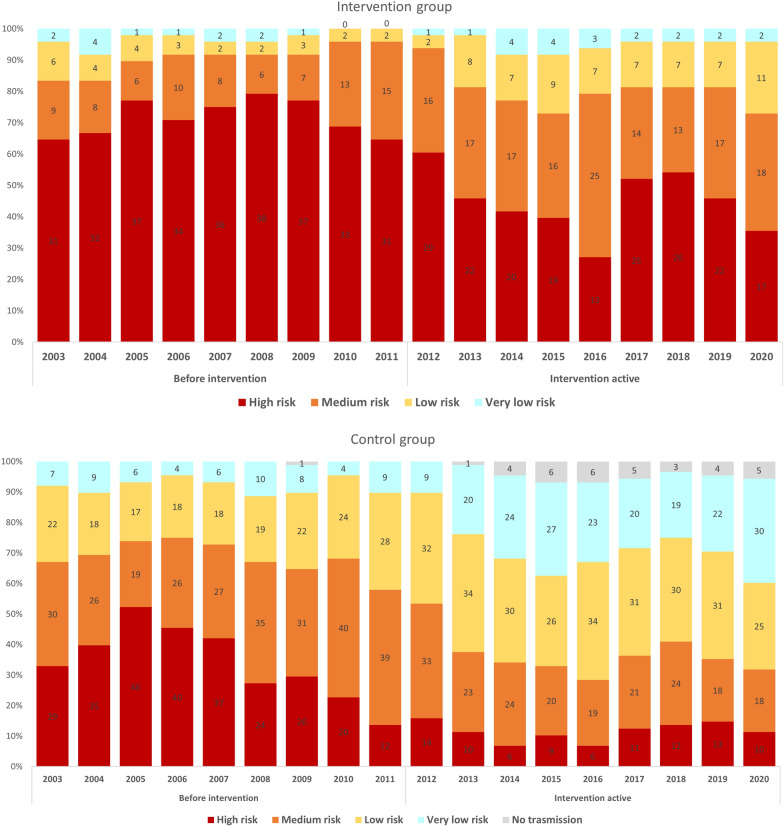
Malaria risk stratification for the 48 supported municipalities in Brazil between 2003 and 2020 (Source: Sivep-Malaria—Ministry of Health)

Moreover, the number of municipalities classified as high-risk (IR > 50 cases per 1000 res.) decreased from 29 (60.4%, N = 48) in the first year of intervention to 17 in 2020 (35.4%, a reduction of 41.3% in the number of high-risk municipalities. The control group registered 14 (15.9%) high-risk municipalities in 2012 and 10 (11.3%) in 2020, a reduction of 28.6% (Fig. 2).

### Interrupted time series analysis

**Fig. 3:**
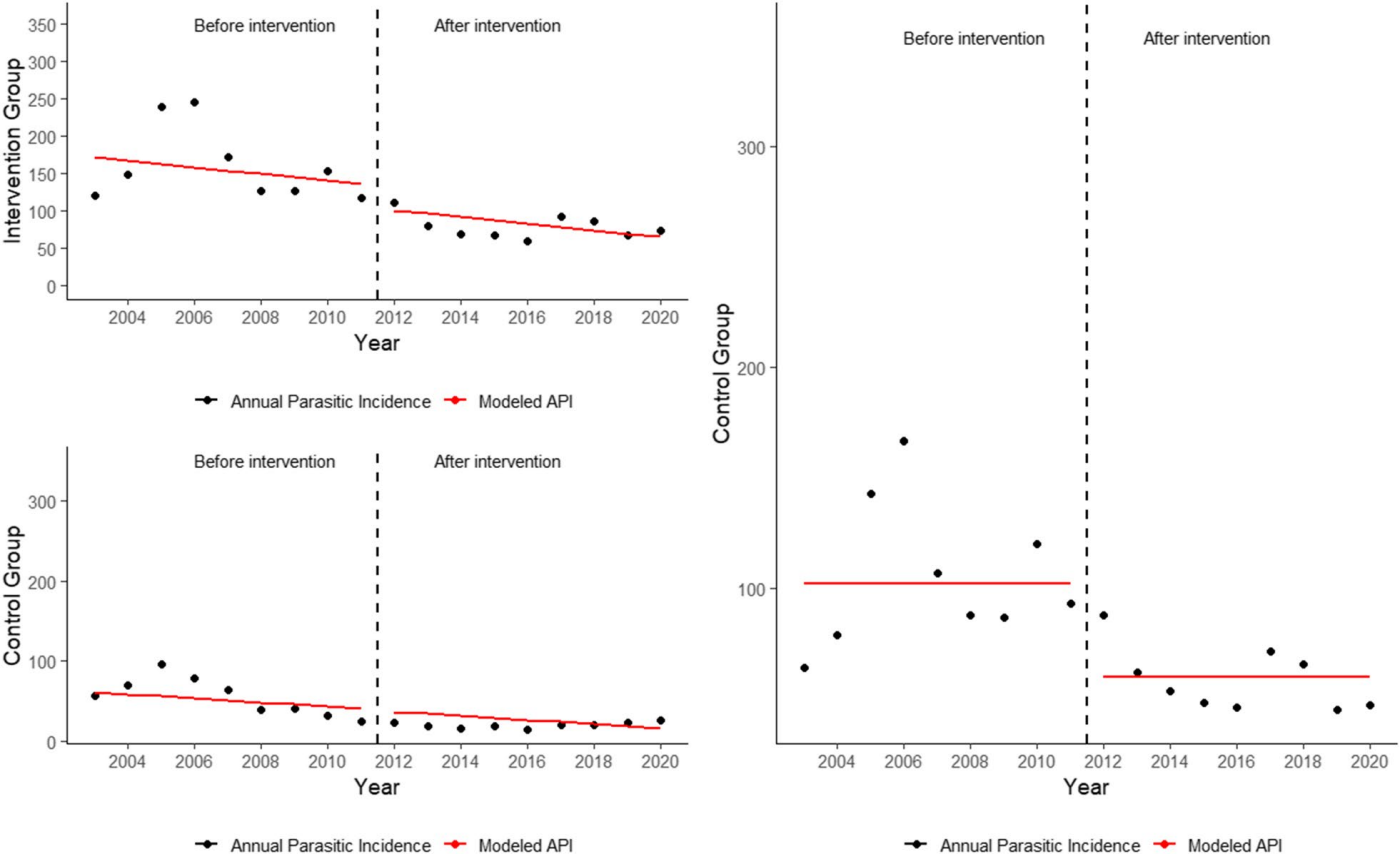
Malaria Annual Parasitic Incidence for intervention and control municipalities, 2003–2020. **A** Intervention and control group PW segmented regression analysis; **B** PW using differencing method between intervention group and control group (Source: Sivep-Malaria—Ministry of Health)

The interrupted time-series analysis comparing the baseline period (2003–2011) with the post-intervention period (2012–2020) showed a significant reduction of − 30.7/1000 res. (95% CI − 103.6; 42.1, p-value: 0.421). Meanwhile, the reduction in the control group was − 1.3/1000 res. (95% CI − 26.7; 24.0, p-value: 0.918). The analysis of the comparison of the intervention group with the control group estimated an annual reduction of − 42.0 cases/1000 res. (95% CI − 70.6; − 13.3, p-value: 0.011) from 2012 to 2020 (Fig. 3).

## Discussion

The cITS analysis presented an effect of − 42.0 cases/1000 reduction during the intervention period in the supported municipalities. Also, there was an indication of effect on the risk classification, in the last year before intervention being implemented there were 31 high-risk municipalities, and in 2020 that number reduced to 17 (− 45.1% reduction). cITS methodologies have been frequently used and indicated for analysing the effects of public health policies and long-term interventions that cover the entire population [25]. An important feature of the analysis is that it quantifies the impact of the intervention at the population level, which often includes herd effects [26, 27].

The analysis of the control group highlights that there was a decreasing trend in the API in the control group before 2012, and a decrease in the number of high-risk and medium-risk municipalities. The reduction in the control shows that there was a geographical reduction already in course in the region. Thus, the additional value of a cITS analysis is the ability to adjust for the underlying secular trend of malaria burden via the addition of a control group, and to subtract the expected reduction and possible confounding variables that influence both control and intervention groups [15]. Therefore, the results shown in Fig. 3 reflect the impact of the intervention in the series without overestimating it. Therefore, the use of that methodology in this paper allowed an adequate visualization of the intervention “Malaria supporters project” effect on the 48 supported municipalities.

Despite the presented analysis, it is still suggested the use of qualitative methodologies, such as Ishikawa diagrams [28] and SWOT matrices [29] to identify possible weaknesses in the intervention activities. That will help on improving the strategy actions and the project resources efficiency use—given the scarce resources for its the execution, possibly increasing its effect.

The positive effects of the Project should not be attributed only to its activities, as there is also the influence of other control strategies that are stimulated by the States and BMoH, such as the distribution of long-lasting insecticidal nets [30, 31], distribution and use of rapid diagnosis tests, opportune and adequate treatment, and health education strategies to the population [4].

The Project has become one of the main strategies for controlling and preventing malaria in Brazil with its activities in the priority municipalities for malaria, and this paper provides a first analysis of the overall impact of the strategy. The PNCM makes continuous efforts for its maintenance and possible expansion, despite challenges such as comparatively lower funding for public health actions from the Brazilian government compared to other countries with public health systems [32, 33].

The scientific literature available on this strategy is yet scarce. So, making dialogue with other authors on the subject is difficult. It is suggested that other studies on the subject be carried out. Recently the BMoH published a material with testimonies on the project [10], those can provide a glimpse of personal opinion on its importance.

The approaches and the project design presented in this study could be replicated to other municipalities that has difficulties in controlling malaria, and it might be useful to be replicated in other countries that struggle with malaria control. Also, it is advised that the project management consider expanding the number of municipalities supported, also aiming at eliminating malaria cases in municipalities with low API. It might also be an effective intervention at state level.

Still, it is necessary to reflect on whether the current structure and activities of the Project are sufficient to achieve the goals of eliminating malaria in Brazil, because as it was stated by Laporta and colleagues in 2022 [34], Brazil has a perspective of non-compliance with those goals by 2035. Also, although the intervention has had a positive impact in controlling malaria API based on this analysis, it is necessary to reinforce that local specificities must be considered in the supporters’ action plan, so they develop their activities with more efficiency.

The necessity to adapt control strategies to different realities in the municipalities of the Amazon region has previously been reported by Lana et al. [35], when they showed that malaria transmission patterns is heterogeneous in the Amazonian cities. So, local adjustments will certainly increase intervention effect. Also, considering that the Brazil’s Amazon region is a study site of several high-quality malaria research projects and scientific communities, it would be beneficial for the intervention effectiveness the development of actions carried out by collaborations between the Project and research and educational institutions.

Therefore, it is crucial to investigate—along with the state and municipalities control programmes—what events in the territory may influence smaller effect in some municipalities, such as illegal gold mining and deforestation. The literature has reported the presence of gold mining activities in the Brazilian Amazon region, especially in the states of Roraima, Amazonas, Amapá, and Pará [36, 37]. These states have several municipalities where the project is present. Then, considering that those activities have influence on malaria endemicity raises, it is possible that gold mining and deforestation activities reduces the intervention effectiveness in those states [38].

## Limitations

The main limitations of this work are related to the imprecision of the provided information regarding the period in which the intervention was active in the intervention group. That resulted in the analyses being carried in annual levels instead of using monthly estimates. That is due some Project’s data lost in 2020, due to cyber-attacks on the Brazilian Ministry of Health network [39]. Additionally, the structure of grouped municipalities (intervention group) did not permit the incorporation of individual times during which each municipality remained without the intervention (information available in Additional file 1). There are inherent limitations to the study method used [40]. The ecological design does not make possible to quantify the impact of the intervention at the level of the municipality, and it is likely that the strength of implementation of the project was heterogeneous across municipalities. Thus, it is interesting that other studies be developed to evaluate the intervention effect at the municipal level. That would allow greater interlocution with the municipal malaria control programmes and consider the specificities of each locality.

While useful and recommended, a major disadvantage of the ITS method is that intervention impact estimates are vulnerable to confounding due to non-intervention bias [16, 40]. If there is a reduction in disease after the intervention is introduced, it is difficult to know whether this is due to the impact of the intervention or other related factors, such as economic development or improved living conditions. Nevertheless, these limitations have been effectively tackled through meticulous control group selection and analysis.

## Conclusions

The results of this study reflect that the project is not a silver bullet to control malaria, but it is an intervention with potential to reduce API in high-risk municipalities. The intervention is a useful tool for malaria control in Brazil and it can have it model replicated in other countries that share similar epidemiological and geographical scenarios such as high endemicity. This is particularly relevant for countries in South America with high endemicity where PAHO support may be available. The model can also be used in programmes to control other infectious and parasitic diseases.

### Supplementary Information

**Additional file 1.** Information access protocols.

**Additional file 2.** Municipalities in intervention and control groups and epidemiological data.

**Additional file 3.** R script with analysis code.

## Data Availability

All aggregated data analysed will be made available in the Additional file 2. Raw epidemiological from Sivep-Malaria data was acquired through formal solicitation to the Brazilian Ministry of Health (protocol #25072.007957/2021-05, and 25072.004310/2023-85).

## Abbreviations

API: Annual Parasitic Incidence
cITS: Controlled interrupted time-series
Fiocruz: The Oswaldo Cruz Foundation
ITS: Interrupted time-series
BMoH: Brazilian Ministry of Health
PAHO: Pan American Health Organization
PNCM: National Programme for the Control and Prevention of Malaria
Sivep-Malaria: Epidemiological Surveillance System for Malaria
SWOT: Strenghts, Weaknesses, Opportunities e Threats
WHO: World Health Organization
