# Supplementary material for: Assessing the impact of the “malaria supporters project” intervention to malaria control in the Brazilian Amazon: an interrupted time-series analysis
Source: Malar J. 2023 Sep 16;22:275. doi: 10.1186/s12936-023-04706-z (PMC10504781; doi:10.1186/s12936-023-04706-z)
Supplement: Supplementary file 1 — Additional file 1. Information access protocols. [file 12936_2023_4706_MOESM1_ESM.pdf]

Additional File I

Protocol # 25072.020758/2021-84

Prezado cidadão, informamos abaixo a resposta à sua solicitação, no prazo da Lei de acesso a informação.

1) **Valores destinados ao financiamento do Projeto Apoiadores Municipais para Prevenção, Controle e Eliminação da malária via *Termo de Cooperação Técnica* - TED entre o Ministério da Saúde e Fundação Oswaldo Cruz - Fiocruz:**

ANO	TED N°	Orçamento TED
2012 à 2013	144	R$ 3.788.449,44
2013 à 2016	152 e 71	R$ 12.799.229,93
2016 à 2018	107	R$ 11.620.788,36
2018 à 2020	107 e 35	R$ 10.000.000,00
2019 à 2022	35	R$ 6.400.000,00

Fonte: Fiocruz e FNS.

2) Quantidade atual de apoiadores atuando no projeto:
24 apoiadores municipais atuando nos seguintes municípios:

Município	UF
Coari	AM
Barcelos	AM
Lábrea	AM
Humaitá	AM
São Gabriel da Cachoeira	AM
Santa Isabel do Rio Negro	AM
Tefé	AM
Ipixuna	AM
Guajará	AM
Mâncio Lima	AC
Rodrigues Alves	AC
Cruzeiro do Sul	AC

Município	UF
Santana	AP
Porto Grande	AP
Mazagão	AP
Oeiras do Pará	PA
Itaituba	PA
Breves	PA
Anajás	PA
Machadinho D'Oeste	RO
Candeias do Jamari	RO
Caracaraí	RR
Cantá	RR
Rorainópolis	RR

Fonte: Projeto Apoiadores Municipais para Prevenção, Controle e Eliminação da Malária com apoio da Fundação Oswaldo Cruz – Fiocruz. Dados coletados em julho de 2021.

3) Distribuição de apoiadores e municípios a partir do início do projeto:
Devido à invasão hacker nos computadores do Ministério da Saúde, ocorrido no ano passado, foram perdidos alguns dados históricos inviabilizando o preenchimento da planilha enviada.
Segue anexo arquivo "Municípios Projeto Malária_TED_02.09.2021.xls" contendo o elenco completo de municípios apoiados de acordo com a fase do projeto.

Estamos à disposição para quaisquer esclarecimentos.

Protocol # 25072.003428/2023-96 (Next page)

Ministério da Saúde
Secretaria de Vigilância em Saúde e Ambiente
Departamento de Doenças Transmissíveis
Coordenação-Geral de Vigilância de Zoonoses e Doenças de Transmissão Vetorial

FORMULÁRIO: RESPOSTA AO CIDADÃO

ASSUNTO:

Solicitação de Acesso à Informação - Plataforma "Fala.BR" - Protocolo NUP nº 25072.003428/2023-96

RESPOSTA:

Prezado Cidadão (ã),

Em resposta ao Pedido de Acesso à Informação, registrado na Plataforma "Fala.BR" sob o protocolo NUP nº 25072.003428/2023-96, envio abaixo respostas:

1. Quando o projeto foi inicialmente construído/desenhado? Quando ele começou a ser implementado? Qual foi a primeira instituição que financiou o projeto e por quanto tempo ela o financiou?

O Projeto Apoiadores Municipais para Prevenção, Controle e Eliminação da Malária (PAMM) é uma estratégia técnica do Programa Nacional de Prevenção, Controle e Eliminação de Malária (PNCM) em parceria com a Fundação Oswaldo Cruz – Fiocruz, que visa primordialmente o fortalecimento da vigilância em saúde no SUS, fortalecendo as ações nos estados e municípios prioritários para a doença no Brasil, surgiu na versão nacional a partir do ano de 2012, após a finalização da cooperação técnica internacional entre o governo brasileiro e o Fundo Global para as ações de malária, ficando formalizado em conferência mundial de saúde o compromisso do Brasil em assumir as estratégias a fim de mitigar o sofrimento da população vulnerável com malária na região amazônica do Brasil.

O projeto tem como pilar a cooperação técnica entre as esferas de governo, sendo federal, estadual e municipal, ou seja, para seu pleno desenvolvimento ambos possuem participação ativa nas atividades e tomada de decisão. Sua formalização se dá via Termo de Execução Descentralizado – TED com a Fundação Oswaldo Cruz – Fiocruz, com financiamento nacional. Os profissionais desenvolvem suas rotinas de trabalho em conjunto com as equipes locais de prevenção, controle e eliminação de malária. A meta é fortalecer o serviço local, repassando para as equipes municipais de saúde uma metodologia de trabalho que possa ser assumida pelas estruturas de vigilância e controle da doença no município, disseminando assim, o conhecimento de forma sustentável.

2. Atualmente, quais são as fontes e como se dá o financiamento do projeto?

O financiamento da estratégia técnica é nacional via Termo de Execução Descentralizado - TED.

3. Como são elencados os municípios que recebem apoio do projeto?

Para a distribuição das vagas no projeto, para além do número absoluto de casos autóctones no município, são levadas em consideração outras análises estratégicas, como: o período em que o município permaneceu como prioritário na região amazônica, bem como as ações de prevenção, controle e eliminação de malária; situação epidemiológica; e capacidade operacional de apoio local ao profissional apoiador e articulação com estados e municípios da região amazônica, sendo priorizadas as áreas de maior endemicidade para malária.

4. Como é feita a interlocução entre Ministério da Saúde e apoiadores?

A interlocução do Projeto Apoiadores Municipais com o Ministério da Saúde, estados e municípios se dá por meio de desenvolvimento e monitoramento de plano de trabalho anual, bem como relatórios técnicos de atividades. Adicionalmente, existem momentos para discussão de estratégias em reuniões online e capacitações presenciais em Brasília.

5. Como é a relação entre a gestão do projeto e as esferas estaduais de saúde? A esfera estadual pode exercer influência no processo de "receber" ou não apoiadores em seus municípios?

A gestão formal do Projeto Apoiadores Municipais fica alocada na Fundação Oswaldo Cruz, esta que possui estreita parceria técnica junto ao Programa Nacional de Prevenção, Controle e Eliminação da Malária do Ministério da Saúde para a definição de lotação dos profissionais apoiadores municipais na região amazônica. Os critérios para lotação de vaga nos estados e municípios estão descritos na resposta 3 deste questionário.

6. Quais são as atividades exercidas pelos apoiadores? Qual a orientação quanto à "forma" que estas atividades devem ser exercidas?

O Projeto Apoiadores Municipais para Prevenção, Controle e Eliminação da Malária (PAMM) é uma estratégia técnica do Programa Nacional de Prevenção, Controle e Eliminação de Malária (PNCM) que visa primordialmente o fortalecimento da vigilância em saúde no SUS, fortalecendo as ações nos estados e municípios prioritários para a doença no Brasil, implementando estratégias para garantir um diagnóstico oportuno e fornecer um tratamento imediato e adequado e intervir com ações de manejo integrado e seletivo de vetores. Este projeto surgiu com a proposta de mitigar um dos desafios encontrados para controlar a malária, que é a redução dos casos de forma sustentável, principalmente em municípios pequenos em áreas remotas da Amazônia. A equipe de apoiadores é formada por profissionais de nível superior na área da saúde, com pós-graduação na área da saúde pública e/ou experiência na área da saúde, capacitada para analisar dados epidemiológicos e orientar de forma estratégica as intervenções de controle da malária, baseados

nos protocolos de trabalho do PNCM, da Coordenação-Geral de Vigilância de Zoonoses e Doenças de Transmissão Vetorial – CGZV do Ministério da Saúde.

As atividades são definidas de acordo com a situação epidemiológica local, tendo como base os eixos temáticos do PNCM com foco na eliminação sustentável da doença.

7. Os apoiadores recebem treinamento antes de irem para os municípios? Se sim, treinamento em quais pontos?

Sim. Todos os profissionais contratados Projeto Apoiadores Municipais para Prevenção, Controle e Eliminação da Malária, após passarem por processo seletivo aberto operacionalizado pela Fiotec, passam por treinamento realizado em parceria com a equipe técnica do PNCM em Brasília tendo como base principal os eixos temáticos da malária (Sistemas de Informação, Vigilância Epidemiológica, Controle Vetorial e Entomologia, Diagnóstico e Tratamento, Educação em Saúde e Mobilização Social, Gestão e Orçamento Público).

8. Os apoiadores possuem acesso aos sistemas de informação de malária? Como o sivep-malaria e vetores-malária? Se sim, qual a justificativa para a concessão deste acesso?

Sim. Porém com acesso restrito que possibilita somente consulta, com estreito monitoramento dos técnicos estaduais e Nacionais, prezando pela Lei Geral de Proteção de Dados.

9. O apoiador atua como técnico de vigilância ou como consultor técnico que representa o Ministério da Saúde?

Não. O profissional apoiador municipal possui seu vínculo formal com a Fiotec, e não representa o Ministério da Saúde.

INFORMAÇÕES COMPLEMENTARES

Ademais, para mais informações sobre a estratégia técnica Projeto Apoiadores Municipais para Prevenção, Controle e Eliminação da Malária (PAMM), poderão ser acessadas no link da publicação abaixo:

https://www.gov.br/saude/pt-br/centrais-de-conteudo/publicacoes/publicacoes-svs/malaria/dez-anos-de-luta-contra-a-malaria-na-amazonia-brasileira-projeto-apoiadores-municipais-para-prevencao-controle-e-eliminacao-da-malaria

(x) Acesso concedido
( ) Acesso negado, justificar a negativa:
[ ] Dados pessoais;
[ ] Informação sigilosa de acordo classificada conforme a Lei nº 12.527/2011;
[ ] Informação sigilosa de acordo com a legislação especifica;
[ ] Pedido desproporcional ou desarrazoado;
[ ] Pedido exige tratamento adicional de dados;

[ ] Pedido genérico;
[ ] Pedido incompreensível;
[ ] Processo decisório em curso.

( ) Acesso parcialmente concedido, justificar:
[ ] Parte da informação contém dados pessoais;
[ ] Parte da informação demandará mais tempo para produção;
[ ] Parte da informação é de competência de outro órgão/entidade;
[ ] Parte da informação é sigilosa de acordo com legislação específica;
[ ] Parte da informação é sigilosa e classificada conforme a Lei nº 12.527/2011;
[ ] Parte da informação é inexistente;
[ ] Parte do pedido é desproporcional ou desarrazoado;[
] Parte do pedido é genérico;
[ ] Parte do pedido é incompreensível;[
] Processo decisório em curso.
( ) Informação inexistente.
( ) Órgão não tem competência para responder sobre o assunto.

O pedido de acesso ou sua resposta contém informações sujeitas à restrição de acesso,
conforme previsto na Lei nº 12.527/2011?

(x) Não ( ) Sim

Área responsável pela resposta
Coordenação Geral de Zoonoses de Doenças de Transmissão Vetorial - CGZV

Atenciosamente,

ANGELICA ESPINOSA BARBOSA MIRANDA

Secretária-Substituta

Secretaria de Vigilância em Saúde e Ambiente

Documento assinado eletronicamente por **Angelica Espinosa Barbosa Miranda**, **Secretário(a) de Vigilância em Saúde e Ambiente substituto(a)**, em 31/01/2023, às 17:55, conforme horário oficial de Brasília, com fundamento no § 3º, do art. 4º, do Decreto nº 10.543, de 13 de novembro de 2020; e art. 8º, da Portaria nº 900 de 31 de Março de 2017.

A autenticidade deste documento pode ser conferida no site http://sei.saude.gov.br/sei/controlador_externo.php?acao=documento_conferir&id_orgao_acesso_externo=0, informando o código verificador **0031575510** e o código CRC **E6A0F330**.

Referência: Processo nº 25072.003428/2023-96 SEI nº 0031575510
